# Foreword: Environmental contaminants and animal health. Proceedings of the Nordic Committee for Veterinary Scientific Cooperation (NKVet)

**DOI:** 10.1186/1751-0147-54-S1-I1

**Published:** 2012-02-24

**Authors:** Raimo Pohjanvirta

**Affiliations:** 1 Chair of the Scientific Programme Committee for the 26th NKVet Symposium, Department of Food Hygiene and Environmental Health, University of Helsinki, Helsinki, Finland

## 

The 26^th^ NKVet (Nordic Committee for Veterinary Scientific Cooperation) Symposium was held in Helsinki, Finland, at 6-7.10.2011. The title of the Symposium was “Environmental Contaminants and Animal Health” and the focus was on the insidious health risks posed to both humans and animals (wild & domestic) by the often ubiquitous synthetic and biological environmental chemicals. The scientific programme consisted of five overview-type keynote speeches along with a total of twelve invited talks on specific topics. The presentations addressed the “classical” but still today highly pertinent environmental toxicants such as dioxins, PCBs and other persistent organic compounds; emerging contaminants such as diverse endocrine disruptors; biological toxins such as mycotoxins; and up-to-date biological assay methodologies available for detection and quantification of these environmental hazards. This special issue of *Acta Veterinaria Scandinavica* contains abstracts or full-length papers of the presentations given in the Symposium. The interested reader should notice that even for most of those presentations of which only the abstract is included here, the original slides (in pdf-format) can be found at the NKVet web site (http://www.nkvet.org/index.php?id=11).

